# Is impaired joint attention present in non-clinical individuals with high autistic traits?

**DOI:** 10.1186/s13229-015-0059-3

**Published:** 2015-12-22

**Authors:** Shuo Zhao, Shota Uono, Sayaka Yoshimura, Motomi Toichi

**Affiliations:** Faculty of Human Health Science, Graduate School of Medicine, Kyoto University, 53 Shogoin Kawahara-cho, Sakyo-ku, Kyoto 606-8507 Japan; International Research Fellow of the Japan Society for the Promotion of Science, Tokyo, Japan; Organization for Promoting Developmental Disorder Research, Kyoto, Japan; Department of Neurodevelopmental Psychiatry, Habilitation and Rehabilitation, Graduate School of Medicine, Kyoto University, Kyoto, Japan

**Keywords:** Autism-spectrum quotient (AQ), Joint attention, Low autistic traits, High autistic traits, Cross-modal

## Abstract

**Background:**

Joint attention skills are impaired in individuals with autism spectrum disorder (ASD). Recently, varying degrees of autistic social attention deficit have been detected in the general population. We investigated gaze-triggered attention in individuals with high and low levels of autistic traits under visual–auditory cross-modal conditions, which are more sensitive to social attention deficits than unimodal paradigms.

**Methods:**

Sixty-six typically developing adults were divided into low- and high-autistic-trait groups according to scores on the autism-spectrum quotient (AQ) questionnaire. We examined gaze-triggered attention under visual–auditory cross-modal conditions. Two sounds (a social voice and a non-social tone) were manipulated as targets to infer the relationship between the cue and the target. Two types of stimulus onset asynchrony (SOA) conditions (a shorter 200-ms SOA and a longer 800-ms SOA) were used to directly test the effect of gaze cues on the detection of a sound target across different temporal intervals.

**Results:**

Individuals with high autistic traits (high-AQ group) did not differ from those with low autistic traits (low-AQ group) with respect to gaze-triggered attention when voices or tones were used as targets under the shorter SOA condition. In contrast, under the longer SOA condition, gaze-triggered attention was not observed in response to tonal targets among individuals in the high-AQ group, whereas it was observed among individuals in the low-AQ group. The results demonstrated that cross-modal gaze-triggered attention is short-lived in individuals with high autistic traits.

**Conclusions:**

This finding provides insight into the cross-modal joint attention function among individuals along the autism spectrum from low autistic traits to ASD and may further our understanding of social behaviours among individuals at different places along the autistic trait continuum.

## Background

One of the key core features of social interactions involves detecting other people’s desires, intentions, and mental states, which helps us to understand their behaviour and the reasons for their actions. The ability to coordinate attention to focus on the same location or event as another person, the phenomenon known as joint attention [1], has been thought to be a precursor of the development of the ability to attribute mental states (i.e. theory of mind). This phenomenon can be achieved when individual B perceives individual A’s direction of attention. Individual B then orients his/her attention to the same object or event. Individuals A and B are now attending to the same object, based on individual B using the attention cues of individual A [2]. In the context of joint attention, developing children typically use the eye gaze of others to make attributions about other people’s cognitive states, such as their intentions, and to speculate about what they want to do [1, 3].

During the past two decades, cognitive psychologists have focused on the social attentional mechanisms of joint attention (e.g. [4–7]). Researchers have commonly investigated gaze-triggered attention using a modification of the Posner cueing paradigm [8]. For instance, in one of the pioneering studies [9], subjects observed an unpredictable, directed eye gaze towards the right or left as a cue, and a letter target was subsequently presented either in the gazed-at or in the opposite direction. Subjects were asked to respond as quickly and as accurately as possible, and their reaction times (RTs) were measured. The RTs for detecting the letter target were faster when it appeared in the same direction as the cues than when it appeared in the opposite direction. This finding indicated that gaze direction reflexively triggers the observer’s attentional orientation. This attentional effect was also found under visual–auditory cross-modal conditions [10] (i.e. using eye gaze as the cue and a tone as the target).

Recently, individual differences in social attention have been identified, particularly in those with autism spectrum disorder (ASD) [11–13]. A lack of joint attention in individuals with ASD has been well documented in the clinical literature [14], and it has attracted attention as an early marker of ASD (e.g. [15–19]; see review in [20]). Furthermore, in our previous study [13], we found that gaze-triggered attention was also impaired under visual–auditory cross-modal conditions in adults with ASD. Additionally, several research studies have suggested that autistic social attention deficiencies may also be found, to different degrees, throughout the general population [21, 22]. It has been suggested that no clear boundary separates normal from psychopathological and that ASD is merely at one extreme end of a continuum [23, 24]. It is, therefore, important to evaluate the distribution of gaze-triggered attention in typical individuals, which might reflect developmental variety in social cognition.

Previous studies [25–27] have demonstrated that autistic traits affect gaze-triggered attention. These studies have generally administered the autism-spectrum quotient (AQ) questionnaire to measure autistic traits in the population at large [23]. For instance, Bayliss and colleagues reported a negative correlation between AQ scores and the magnitude of the gaze-cueing effect [25] and revealed that the effect of target context (i.e. scrambled vs. normal face) on gaze-triggered attention differed as a function of an individual’s placement on the autistic spectrum. In particular, a greater gaze-triggered attention effect was observed for a scrambled face context compared with a normal face context among individuals with a high level of autistic traits, suggesting that this bias was related to the level of attention to detail [26]. Moreover, Hudson et al. [27] found a smaller cueing effect in response to gaze in antisocial individuals than in prosocial individuals within a low-AQ group; the effect was equal in both cue within the high-AQ group. All these studies used visual cues and visual targets under a unimodal condition. However, real life includes various environmental stimuli, including sounds. We need to constantly coordinate our attention with that of others by using cues and targets that belong to different modalities; therefore, it is also necessary to investigate the underlying mechanisms of how we modulate the effects of gaze-triggered attention under cross-modal conditions and to examine how these vary with autism traits and relate to a diagnosis of autism.

We attempted to extend the previous research in the following respects. First, the experimental paradigms used in previous studies [25–27] have not been applied to gaze-triggered attention in ASD individuals, resulting in a lack of evidence of the overall variation in gaze-triggered attention associated with autistic traits, including ASD. Second, most of the previous studies found intact gaze-triggered attention when visual cues and targets were used under unimodal conditions ([28–30]; for a review, see [31]). In contrast, our previous study [13] clearly showed that gaze-triggered attention was impaired in individuals with ASD under cross-modal conditions. Gaze-triggered attention was impaired in individuals with ASD when the cue–target relationship was weak (i.e. a social gaze cue and a non-social tone target), whereas it was unimpaired when there was a strong cue–target relationship (i.e. a social gaze cue and a social voice target). Hence, to bridge the gap between our understanding of the gaze-triggered attention of typically developing individuals and that of the gaze-triggered attention of individuals with a diagnosis of ASD, this paper focuses on the gaze-triggered attention of individuals with autistic traits in response to a visual cue and an auditory target (i.e. cross-modal conditions).

In this study, we first examined gaze-triggered attention under visual–auditory cross-modal conditions. Two sounds (a social voice and a non-social tone) were manipulated as targets to infer the relationship between the cue and target. The previous study [13] indicated that the effect of gaze cues on individuals with ASD could be mediated by different stimulus onset asynchronies (SOAs) between the auditory target and the gaze cue (i.e. gaze-triggered attention was observed when the target was a voice at a shorter, 200-ms, SOA, whereas the effect was not observed at a longer, 800-ms, SOA). Thus, to test the effect of gaze cues on the detection of a sound target, we used two types of SOA. Participants were asked to identify the direction of an auditory target as accurately and rapidly as possible following a gaze cue. All participants were divided into low- or high-autistic-trait groups according to AQ score. Based on the previous study [13], we hypothesised that individuals with high autistic traits would be impaired with respect to gaze-triggered attention when the cue–target relationship was weak (i.e. a social gaze cue and a non-social tone target). In contrast, we predicted that the gaze-triggered attention of individuals with low autistic traits would be intact even when the cue–target relationship was weak. Taken together, the aims of the study were as follows: (1) to investigate whether visual–auditory cross-modal gaze-triggered attention is impaired in individuals with high autistic traits and (2) to examine whether the visual–auditory cross-modal gaze-triggered attention of these individuals is impaired when the cue–target relationship is weak (i.e. a social gaze cue and a non-social tone target).

## Methods

### Ethics statement

The experimental procedures were approved by the local ethics committee of the Graduate School and Faculty of Medicine at Kyoto University. There were no foreseeable risks to the participants, and no personally identifying information was collected. Participants provided background information and gave written informed consent. The procedures complied with the ethical standard of the 1964 Declaration of Helsinki regarding the treatment of human participants in research.

### Participants

Sixty-six naïve participants (mean age = 20.67 ± 0.966 standard deviation (*SD*) years; 41 women, 25 men) recruited from Kyoto University participated in the experiment for payment. All participants were right-handed, as assessed by the Edinburgh Handedness Inventory [32], and had normal or corrected-to-normal visual and auditory acuity.

### Apparatus

Stimulus presentation and data acquisition were controlled using Presentation (NeuroBehavioral Systems) on a Windows computer. Stimuli were presented on a 19-in. monitor (Dell; screen resolution 1024 × 768 pixels; refresh rate 60 Hz). The distance between the monitor and the participants was fixed at approximately 57 cm using a headrest. All auditory stimuli were presented through headphones.

### Stimuli

Consistent with the previous study, the same neutral face of a female model (MO) was selected from Ekman and Friesen [33]. The gaze direction was then manipulated. The irises and pupils of the eyes were cut from the original photographs and pasted to fit over the right or left side of the eyes using Photoshop 5.0 (Adobe). We cropped the photographs in an ellipse 8.3° wide and 12.1° high to exclude hair and background.

We used two types of auditory stimuli as targets. One was sampled from a native Japanese woman: an /i/ voice sound (F0 frequency of 300 Hz, 80 dB sound pressure level (SPL)), which is similar to the /iy/ sound in English. The other was a pure tone of a voice with a frequency similar to F0 (300 Hz, 80 dB SPL), which was produced using Audacity V1.3.13 software (AudacityStore.com). Both auditory targets had a duration of 150 ms.

### Design

The experiment was constructed as a four-factorial, mixed randomised-repeated design, with auditory (voice sound or tone sound), validity (valid, invalid), and SOA (200, 800 ms) as repeated factors and group (low- or high-AQ group) as the randomised factor.

### Measures

We used a Japanese version of the AQ [34], which is a 50-item self-rated scale designed to measure each of five domains of interest: social skill, communication, imagination, attention to detail, and attention-switching. Ten statements were used to measure each of the assessed traits, and participants were asked whether they ‘strongly agree’, ‘slightly agree’, ‘slightly disagree’, or ‘strongly disagree’ with each statement. Participants with a high score in the AQ possessed a greater number of autistic traits. The AQ score has been shown to have good test–retest reliability, good internal consistency, and acceptably high sensitivity and specificity [23]. In this sample, the mean AQ score was 19.44 ± 7.06 *SD* of 50. Based on previous research [27], participants scoring lower than the median AQ score of 18.5 were assigned to the low-AQ group (*M* = 13.64, *SD* = 3.92, range = 5–18, *N* = 33), whereas those scoring higher than the median AQ score of 18.5 were assigned to the high-AQ group (*M* = 25.24, *SD* = 4.05, range = 19–35, *N* = 33). Kurita et al. [34] reported a cut-off of 32 for screening adults with high-functioning pervasive developmental disorders (PDD) using a Japanese version of the AQ (AQ-J). In this study, we considered individuals in the high-AQ group to have milder autistic traits than those diagnosed with ASD. The gender ratio in the two groups did not significantly differ (12 men and 21 women in the low-AQ group, 13 men and 20 women in the high-AQ group, Fisher’s exact test, *p* > 0.1).

### Procedure

**Fig. 1:**
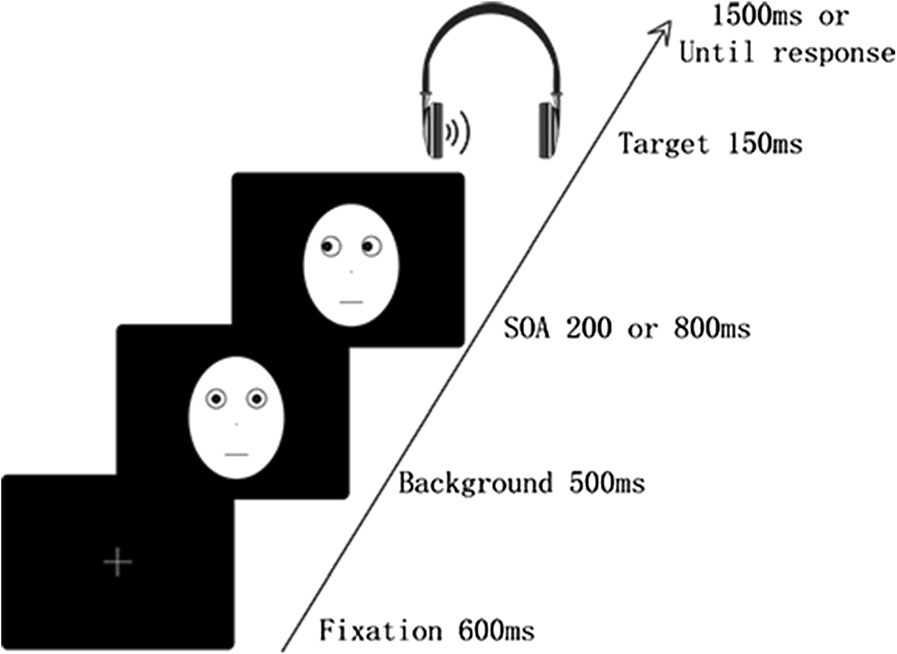
Illustrations of stimulus presentations. Actual stimuli were photographs of faces (see Figure 1 in [33])

We used the same experimental paradigm as in our previous study [13] (see Fig. 1). For each trial, a fixation cross point was first presented in the centre of the screen for 600 ms. A neutral face with a straight gaze was then presented at this location as a background. After 500 ms, a neutral facial cue with the eye gaze directed right or left was presented in the centre of the screen. The SOA between the auditory target and gaze cue was 200 or 800 ms. The SOA condition was determined by randomising each auditory target condition to exclude an effect specific to a sequence of SOA conditions. Subsequently, an auditory stimulus target (voice sound or tone sound) was presented in the left or right ear through headphones for 150 ms. Participants were asked to communicate as quickly and exactly as possible whether the target was presented to the left or to the right ear by pressing the corresponding key on the switch key using the index or middle finger of their dominant hand, respectively. Response time (RT) was measured in each trial. The gaze cue remained visible until the response or until 1,500 ms had elapsed. The targets appeared randomly on the same or opposite side as the gaze direction when the eyes looked left or right. If participants could not respond in a trial, the data were excluded as incorrect. The target appeared in the cued location in 50 % of the trials. Participants were told that the cues did not predict the target location and were instructed to fix on the centre of the screen in each trial.

The experiment consisted of eight blocks of 52 trials, including 32 catch trials in which the target did not appear. Forty-eight trials were performed under each condition. Each condition was presented in a pseudorandom order. Participants were allowed to rest between blocks. Fifty-two practice trials preceded the experimental trials. At the end of the experiment, all participants completed the AQ questionnaire.

### Analysis

The data were analysed using IBM’s SPSS Statistics software. Incorrect responses and responses of <150 ms or >1000 ms (0.24 % of the trials) were excluded from the RT analysis. The mean RT under each condition was calculated for each participant. First, trials with RTs faster than 150 ms or slower than 1000 ms (low-AQ group: 0.17 % of trials, high-AQ group: 0.32 % of trials) and those with incorrect responses (low-AQ group: 1.77 % of trials, high-AQ group: 1.96 % of trials) were excluded from the RT analysis.

Then, the mean RT of all participants was log_10_-transformed and submitted to a four-way repeated-measures analysis of variance (ANOVA) with validity (valid, invalid), auditory target (voice, tone), and SOA (200, 800 ms) as within-participant factors, and group (low AQ, high AQ) as the between-participant factor. Data from significant interactions were analysed separately, using three-way ANOVAs for 200 and 800 ms SOA. To examine whether two three-way ANOVAs were significant for the interaction, if present, follow-up simple effect analyses were conducted. Furthermore, a preliminary analysis of covariance (ANCOVA), using participants’ gender as a covariate, revealed that gender did not affect the group × validity × target × SOA interaction.

## Results

### Cueing effects on accuracy

There was no significant difference in the error rates of the low- and high-AQ groups (*F* (1, 64) = 0.14, *p* = 0.71, *η*p2 = 0.002). We found a significant main effect of validity (*F* (1, 64) = 30.19, *p* < 0.001, *η*p2 = 0.32), with fewer incorrect responses under the valid compared with the invalid condition (1.3 vs. 2.5 %) and of SOA (*F* (1, 64) = 33.32, *p* < 0.001, *η*p2 = 0.34) and with more incorrect responses under the shorter compared with the longer SOA condition (2.4 vs. 1.4 %); we also found a significant interaction of validity × SOA (*F* (1, 64) = 11.34, *p* = 0.001, *η*p2 = 0.015), indicating fewer incorrect responses in valid compared with invalid ones under the shorter SOA (1.3 vs. 3.4 %) but not under the longer SOA (1.2 vs. 1.6 %) condition.

### Cueing effects on reaction times

We first investigated whether the data set was well-modelled by a normal distribution in all of the conditions. However, when using tone and validity at the 800 ms SOA condition, the data were not normally distributed (Shapiro–Wilk: *p* = 0.04). Therefore, the mean RT data were logarithmically transformed and analysed using a four-way ANOVA. We conducted a two-group (low-, high-AQ group) × two-target (voice, tone) × two-validity (valid, invalid) × two-SOA (200, 800 ms) analysis. The ANOVA revealed a significant main effect of validity (*F* (1, 64) = 51.65, *p* < 0.001, *η*p2 = 0.45), with faster responses under the valid than the invalid condition. There was no significant main effect of SOA (*F* (1, 64) = 1.02, *p* = 0.32, *η*p2 = 0.016), target (*F* (1, 64) = 3.74, *p* = 0.06, *η*p2 = 0.06), or group (*F* (1, 64) = 0.30, *p* = 0.58, *η*p2 = 0.005), suggesting that the perceptual salience of the two types of stimuli, and the perceptual response in each group, did not differ. Significant interactions of validity × SOA (*F* (1, 64) = 97.34, *p* < 0.001, *η*p2 = 0.60), target × validity × SOA (*F* (1, 64) = 7.76, *p* = 0.007, *η*p2 = 0.108), and group × target × validity × SOA (*F* (1, 64) = 4.35, *p* = 0.04, *η*p2 = 0.06) were also found.

#### No difference of gaze-triggered attention under the short (200 ms) SOA condition between the low- and high-AQ groups

**Fig. 2:**
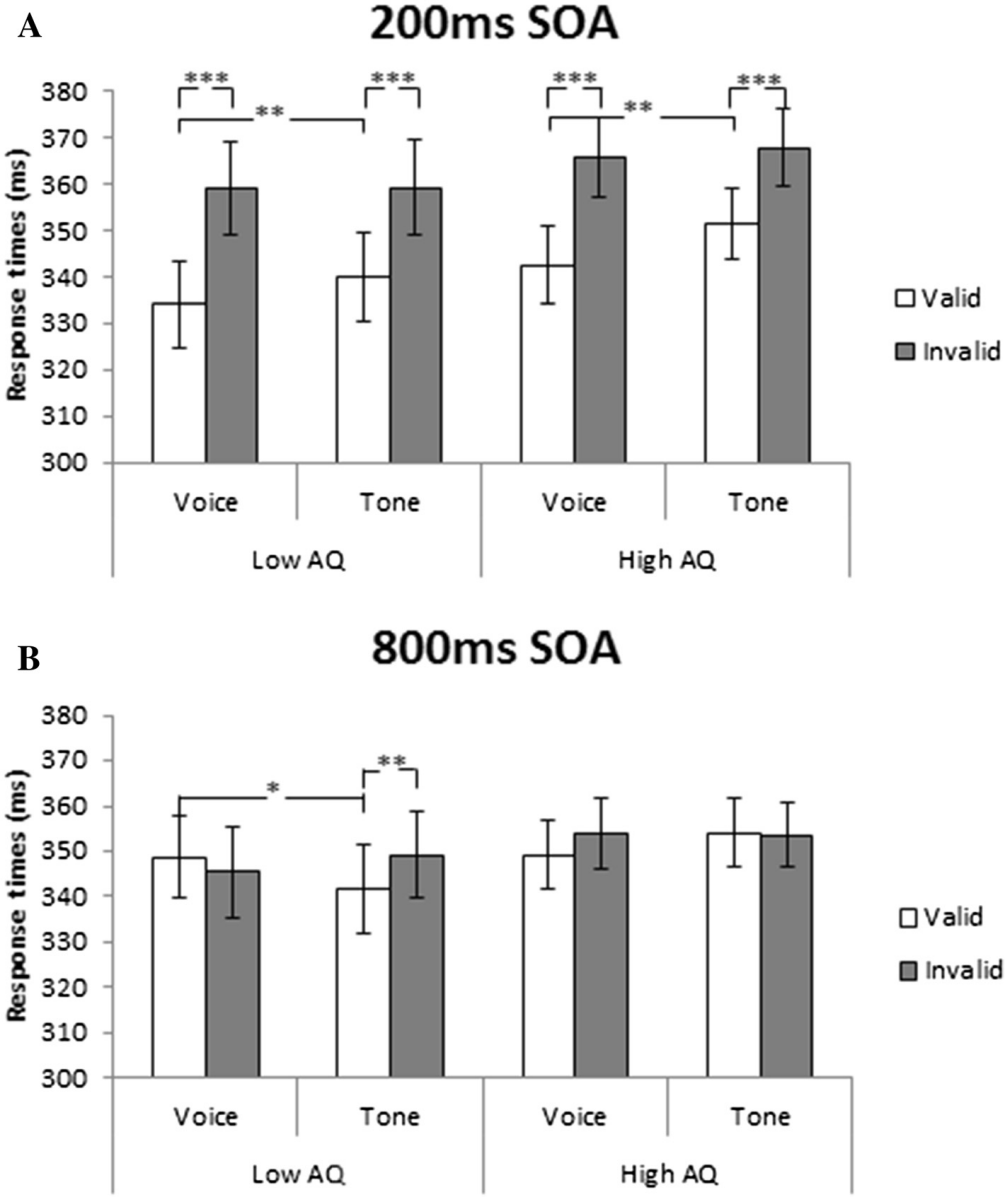
Effect of gaze direction on response times (RTs) under each condition with 200 ms (**a**) and 800 ms (**b**) SOA. *Error bars* represent standard errors of the mean (SEMs). ***p < 0.001, **p < 0.01, *p < 0.05

Because we found significant interactions among the four factors, the data were analysed separately for the 200- and 800-ms SOA conditions. A three-way ANOVA of the RT (logarithmically transformed) data under the 200-ms SOA condition (Fig. 2a, Table 1a) revealed a significant main effect of validity (*F* (1, 64) = 100.85, *p* < 0.001), with faster responses under valid than invalid conditions, and a marginally significant effect of target (*F* (1, 64) = 8.18, *p* = 0.06), with faster responses under the voice than the tone condition; however, there was no significant effect involving group (*F* (1, 64) = 0.42, *p* = 0.52). Interestingly, there was a significant interaction of target × validity (F (1, 64) = 5.40, *p* = 0.02). A post hoc test revealed a significant validity effect (both of *p* < 0.001) using voice and tone as targets, indicating that gaze-triggered attention was elicited using voice and tone as targets in both the low- and high-AQ groups. Furthermore, a significant difference was found with the valid condition between voice and tone as targets across groups (*p* = 0.01), suggesting that gaze-triggered attention was elicited more rapidly when using voice rather than tone targets. These results reveal that gaze-triggered attention to a voice target was enhanced in both the low- and high-AQ groups under the shorter SOA condition.

**Table 1:** Mean low- and high-AQ group response times (ms) to auditory targets according to validity and stimulus onset asynchrony

Auditory target	Voice		Tone	
	Valid	Invalid	Valid	Invalid
Stimulus onset asynchrony				
(a) 200 ms (SEM)				
Low-AQ group	333.9 (8.3)	358.8 (8.4)	339.6 (7.6)	359.1 (8.4)
High-AQ group	342.4 (9.3)	365.5 (9.9)	351.3 (9.5)	367.6 (10.1)
(b) 800 ms (SEM)				
Low-AQ group	348.6 (7.6)	345.4 (7.8)	341.7 (7.6)	349.2 (7.1)
High-AQ group	349.2 (9.1)	354.0 (9.9)	354.0 (9.7)	353.6 (9.6)

#### Impaired gaze-triggered attention under the long (800 ms) SOA condition in the high-AQ group

In terms of the 800-ms SOA condition (Fig. 2b, Table 1b), a three-way ANOVA of the RT (logarithmically transformed) data revealed no significant main effect with respect to target (*F* (1, 64) = 0.05), validity (*F* (1, 64) = 1.77), or group (*F* (1, 64) = 0.18) (*p* > 0.1 for all). More importantly, there was a significant interaction of target × validity × group (*F* (1, 64) = 7.07, *p* = 0.01). A post hoc test revealed a significant validity effect using tone as the target in participants with low-AQ scores (*p* = 0.04), indicating that gaze-triggered attention was elicited when using tones as targets. Furthermore, a significant difference was found between voice and tone targets under the valid condition in the low-AQ group (*p* = 0.03), suggesting that inhibition of return (IOR) was faster for voice than tone targets in participants with low-AQ scores. In contrast, no significant difference was found for validity or targets in participants with high-AQ scores (all *p* > 0.1). These results showed that gaze-triggered attention was observed under the longer SOA condition in participants with low-AQ scores, but not in participants with high-AQ scores.

## Discussion

This study manipulated gaze-triggered attention using sound targets (i.e. voice and tone) and SOA (200 and 800 ms) under visual–auditory cross-modal conditions to examine whether this capacity is related to the extent of autistic-like traits, measured with the AQ. The gaze-triggered attention of individuals with high levels of autistic traits (the high-AQ group) did not differ from that of individuals with low levels of autistic traits when voice or tone was used as a target under the shorter SOA condition. In particular, voice but not tone facilitated gaze-triggered attention in both low- and high-AQ groups. In contrast, the low-AQ but not the high-AQ group exhibited significant gaze-triggered attention to a tone target under the longer SOA condition. This result suggests that the gaze-triggered attention of individuals with high autistic traits was short-lived in response to a sound stimulus.

### Similar gaze-triggered attention between individuals with low and high autistic traits at the shorter SOA

Previous findings [13] were replicated in this study, as we observed that a shorter SOA facilitated gaze-triggered attention more rapidly to voice than tone targets in individuals both low and high in autistic traits. Previous studies of normal individuals [35–40] demonstrated that gaze-triggered attention is facilitated when the cue and target are congruent (e.g. a happy face as a cue and a pleasant infant as the target). Consistent with these previous studies, a higher level of gaze-triggered attention was found in both groups under the shorter SOA condition when the cue–target relationship was congruent (i.e. a social cue and a social target) than when it was incongruent (i.e. a social cue and a non-social target). Although our previous study of individuals with ASD [13] also demonstrated the same pattern of gaze-triggered attention under the shorter SOA condition, it did not reveal a significant gaze-cueing effect when there was a weak cue–target relationship (i.e. a social cue and a non-social target). Based on these findings, the results suggest that gaze-triggered attention is modulated by the cue–target relationship in individuals across the autism spectrum and that it is intact in normal participants, even when the cue–target relationship is weak.

### Impairment in gaze-triggered attention in individuals with high autistic traits at the longer SOA

Under the longer SOA condition, individuals with low autistic traits showed a significant gaze-cueing effect in response to a tone but not to a voice target. Previous studies [13, 41] have suggested that IOR was modulated by the cue–target relationship in typical individuals. These studies found that an IOR effect occurred earlier during the time course of SOA when the contextual cue–target relationship was strong (i.e. a social cue and a social target) than when it was weak (i.e. a social cue and a non-social target). Consistent with these findings [13, 41], the present study observed a faster IOR of gaze-triggered attention in individuals with low autistic traits when the cue–target relationship was strong (i.e. congruence between the social gaze cue and the social voice target) than when the cue–target relationship was weak (i.e. incongruence between the social gaze cue and the non-social tone target) under the longer SOA conditions. In contrast, in individuals with high autistic traits, gaze-triggered attention was not observed in response to voice and tone targets at the longer SOA. These results suggest that individuals with high levels of autistic traits do not demonstrate contextual modulation of the IOR effect and that the cueing effect for both kinds of targets is diminished at the intermediate SOA. Moreover, a previous study [42] showed that the IOR effect occurred earlier in individuals with ASD than in normal individuals when using non-predictive target-peripheral cues during the time course of SOA; the IOR effect was present at the shorter SOA (300 ms) in individuals with ASD and at the longer SOA (500–700 ms) in normal individuals. Based on this study, the impairment in gaze-triggered attention to tone targets at the longer SOA might also be caused by the early-onset appearance of IOR in individuals with high autistic traits.

Zhao et al. [13] proposed that gaze-triggered attention is likely to be impaired in individuals with ASD when the contextual relationship between a cue and a target is weak (i.e. a gaze cue and a tone target); gaze-triggered attention was impaired for tone targets during shorter and longer (200 and 800 ms) SOAs. Whereas participants with low autistic traits showed significant gaze-triggered attention to tone targets across the time course, participants with high autistic traits, who did not meet criteria for ASD, showed impaired gaze-triggered attention to tone targets at only the longer SOA. In addition to increasing with the degree of severity of autistic traits, gaze-triggered attention was also impaired by the strength of the contextual relationship between cue and target. Given that an identical paradigm (with fewer trials) was also implemented in our previous study [13], a quantified variability of the gaze-cueing effect (i.e. the difference in score between the valid and invalid conditions) to tone targets was observed along the autistic spectrum; scores for the group with high autistic traits (7.9 ms ± 2.9 SD) were intermediary to the scores for the low autistic traits (13.5 ms ± 2.9 SD) and ASD (1.3 ms ± 3.9 SD) groups. Thus, we suggest that the degree of impairment in gaze-triggered attention varies with autistic traits. These findings might contribute to understanding the continuum that includes individuals with low and high autistic traits and diagnoses of ASD in terms of cross-modal joint attention and might improve our understanding of social behaviours among individuals along the autistic spectrum.

### Implications of the development of social cognition

The pattern of gaze-triggered attention shown by participants with low and high levels of autistic traits differed. This finding might reflect developmental variety in social cognition within the general population. If differences regarding the processing of social information emerged in infancy, they may continue to affect cognitive abilities into adulthood. Importantly, the attention with gaze directional cues to a specific target shown by individuals with high autistic traits did not endure. Mundy and colleagues [43–45] proposed that joint attention enhances the processing of information, including others’ internal states and attended objects, and that individuals with autism do not receive the benefits of enhanced information processing through joint attention with others. Consistent with the prediction of this model, previous studies have found that typically developing individuals show a greater resistance to IOR that emerges in social (i.e. 800 ms) than in non-social directional cueing (i.e. 300 ms) [46, 47]; this suggests that information processing of an attended object is more effective with social directional cues than with non-social directional cues. However, the current study speculated on an early-onset of IOR to social cues, even in typically developing individuals with high autistic traits. Another study found that, under a joint attention context, gaze allocation to another’s eye gaze was higher for directed targets than non-directed targets in individuals with low levels of autistic traits; no differences were observed in individuals with high levels of autistic traits [48]. Based on the evidence, information processing of attended objects under the context of joint attention may be less enhanced in typically developing individuals with high autistic traits than in those with low autistic traits. Furthermore, given that joint attention is a precursor to theory of mind [49], impaired gaze-triggered attention might impede and differentially affect the development of the ability to understand others’ mental states (beliefs, desires, intentions, imagination, emotion, etc.) of individuals with high autistic traits. Hence, the findings of the current study may improve our understanding of the social behaviours of people who have high autistic traits without a diagnosis of ASD.

### Limitations in the current study

First, given that the AQ is a continuous measure, it is a very interesting question whether there is a correlation between AQ score and gaze-cueing effect. However, we did not find a significant correlation with AQ scores in the magnitude of gaze-cueing effect (all *p* > 0.1). One possible explanation is that in individuals with intermediate autistic traits, gaze-cueing effect may be modulated less by autistic traits and more by general individual differences in, for example, IQ or motor performance. These data were not collected in our study, which presents a limitation of the findings. Second, the stimulus presentation consisted of a passive viewing paradigm. However, in real life, social interactions are typically characterised by reciprocity and interdependence of behaviours rather than simple passive stimulus-response patterns. Recent developments in social cognition research have moved towards using socially responsive agents [50–53] to investigate social attention under an ecologically valid context. Future research may benefit from investigations of the initiation and response to gaze-triggered attention in individuals along the autistic trait spectrum using interactive-response patterns such as gaze-contingent stimuli.

## Conclusions

Our results demonstrated that gaze-triggered attention was impaired in response to tone targets at the longer SOA in individuals with high autistic traits, whereas their gaze-triggered attention to voice and tone targets was intact at the shorter SOA, indicating that gaze-triggered attention was short-lived and reduced in individuals with high autistic traits. More generally, our findings highlight the importance of the overall variation in gaze-triggered attention among those with autistic traits, including individuals with ASD, and underscores the fact that the degree of impairment in gaze-triggered attention varies as a function of autistic traits. Thus, this research may offer a new perspective on the social cognition of individuals along the autistic spectrum and help future researchers carefully consider their recruitment strategy, particularly when investigating certain gaze-triggered attention tasks.

## Abbreviations

AQ: autism-spectrum quotient
ASD: autism spectrum disorder
